# Multilocus sequence types of clinical *Burkholderia pseudomallei* isolates from peninsular Malaysia and their associations with disease outcomes

**DOI:** 10.1186/s12879-017-2912-9

**Published:** 2018-01-02

**Authors:** Abdel Rahman Zueter, Zaidah Abdul Rahman, Mahmoud Abumarzouq, Azian Harun

**Affiliations:** 1 Department of Medical Laboratory Sciences, Faculty of Allied Health Sciences, The Hashemite University, Zarqa, Jordan; 2 Department of Medical Microbiology and Parasitology, School of Medical Sciences, Universiti Sains Malaysia, 16150 Kubang Kerian, Kelantan Malaysia; 3 Department of Orthopedic, School of Medical Sciences, Universiti Sains Malaysia, 16150 Kubang Kerian, Kelantan Malaysia

**Keywords:** Burkholderia pseudomallei, Melioidosis, MLST, Sequence type, Risk

## Abstract

**Background:**

Previous studies on the *Burkholderia pseudomallei* genetic diversity among clinical isolates from melioidosis-endemic areas have identified genetic factors contributing to differential virulence. Although it has been ruled out in Australian and Thai *B. pseudomallei* populations, it remains unclear whether *B. pseudomallei* sequence types (STs) correlate with disease in Malaysian patients with melioidosis.

**Methods:**

In this study, multi-locus sequence typing (MLST) was performed on clinical *B. pseudomallei* isolates collected from Kelantan state of Malaysia, patients’ clinical data were reviewed and then genotype-risk correlations were investigated.

**Results:**

Genotyping of 83 *B. pseudomallei* isolates revealed 32 different STs, of which 13(40%) were novel. The frequencies of the STs among the 83 isolates ranged from 1 to 12 observations, and ST54, ST371 and ST289 were predominant. All non-novel STs reported in this study have also been identified in other Asian countries. Based on the MLST data analysis, the phylogenetic tree showed clustering of the STs with each other, as well as with the STs from Southeast Asia and China. No evidence for associations between any of *B. pseudomallei* STs and clinical melioidosis presentation was detected. In addition, the bacterial genotype clusters in relation with each clinical outcome were statistically insignificant, and no risk estimate was reported. This study has expanded the data for *B. pseudomallei* on MLST database map and provided insights into the molecular epidemiology of melioidosis in Peninsular Malaysia.

**Conclusion:**

This study concurs with previous reports concluding that infecting strain type plays no role in determining disease presentation.

## Background

*Burkholderia pseudomallei* (agent of melioidosis) is acquired by inoculation, inhalation and ingestion routes. It causes wide spectrum clinical presentations; particularly in patients with diabetes mellitus [1]. Marked heterogeneity is observed in the clinical presentation and disease severity among patients. The most severe manifestations of melioidosis are pneumonia and severe sepsis [2]. Melioidosis predominates in Southeast Asia and northern Australia [3, 4]. Regional variations in melioidosis signs and symptoms have been reported and prostatic abscess and encephalomyelitis are common in Australians. Parotid abscesses and hepatosplenic suppuration presentations have been described frequently in Thailand [5–7]. There is good evidence that certain *B. pseudomallei* genes contribute to different clinical presentations between Asia and Australia; in particular, the bimABm gene, which has been strongly associated with neurological melioidosis [8]. The reason behind this diversity remains unclear, but it may be due to host, bacterial, or environmental factors [2].

The study of molecular epidemiology has provided additional details regarding bacterial diversity and distribution [3]. Commonly applied *B. pseudomallei* molecular epidemiology procedures include pulsed-field gel electrophoresis (PFGE) [9, 10], random amplification of polymorphic DNA (RAPD) [11], ribotyping [12] and whole genome sequencing [13]. Multi-locus sequence typing (MLST) is another molecular approach that simplifies the exchange of local and global inter-laboratory genotyping data [14]. The discriminating ability of MLST between different *B. pseudomallei* genotypes was evaluated previously by comparison with PFGE and similar results were reported [2].

Typing of *B. pseudomallei* using MLST scheme is useful to explore sequence types (STs) in particular populations [15], predict the distribution of bacterial STs in a given geographical area [16], track the source of melioidosis outbreaks [17] and define whether recurrent melioidosis is due to a relapse of the same bacterial ST or reinfection with a different ST [18].

The *B. pseudomallei* STs must be studied in Peninsular Malaysia to understand the population genetics in this region and to determine the distribution and frequency of genotype associations in melioidosis cases. MLST was applied for this purpose. According to a literature database search, no national or local project has applied MLST to *B. pseudomallei* isolates collected from Peninsular Malaysia. However, some genotyping studies have used pooled isolates from different regions of Southeast Asia, including Malaysia [19]. Thus, this is the first study to compare STs of clinical isolates from Peninsular Malaysia and to determine whether particular STs are associated with particular clinical outcomes.

## Methods

### *B. pseudomallei* isolates source

Clinical samples were collected, bacteria were isolated *and B. pseudomallei* was identified and archived as part of routine diagnostics in accordance to the standard protocol at the Medical Microbiology & Parasitology Laboratory at the Hospital Universiti Sains Malaysia (HUSM). Only a single clinical isolate from each patient was obtained to preserve the assumption of independence of observations and to avoid repetition.

### Multi-locus sequence typing

MLST was performed as described previously by Godoy et al. [19]. New allelic profiles were confirmed by a repeated MLST procedure. Novel STs were assigned new allelic profile numbers and were submitted, with the isolate information to the *Burkholderia* MLST database (http://pubmlst.org/bpseudomallei/). The submission process was completed from November 2012 to April 2015.

#### Phylogenetic analysis

Basic statistical quantities such as number of alleles, number of variable sites per allele, number and frequency of single nucleotides polymorphism (SNPs) in each locus and the nucleotide sequence diversity rate were calculated and displayed using functional options in molecular evolutionary genetics analysis version-6 (MEGA 6) software [20]. Relatedness among isolates was estimated based on two principles: differences in allelic profiles using eBURST v7 [21, 22]. and differences in the concatenated sequence of alleles at all loci using MEGA 6 software.

All STs were uploaded into eBURST v7 software to display the relatedness among the isolates obtained in this study, as well as among *B. pseudomallei* of the historical collection from different regions in Malaysia. Three population snapshot diagrams were generated: the first diagram displayed the relatedness of the novel and existing STs reported in this study. The second and third diagrams were made for STs of the MLST database for Malaysia before and after the addition of STs obtained from this study to display the significant changes on the full-size Malaysian MLST database population snapshot.

Sequences of every allelic profile were joined in the order of loci used to define the allelic profile to achieve a concatenated sequence of 3399 bp. The topology and grouping of all STs retrieved from this study were displayed on the constructed bootstrapped phylogenetic trees using Unweighted Pair Group Method with Arithmetic average (UPGMA) method in MEG 6 software. STs obtained from this study were analyzed with selected 88 STs representing Malaysia and regional endemic countries including India, China, Singapore, Indonesia, Laos, Vietnam, Philippines, Bangladesh and Thailand.

### Genotype-disease associations

Patient records were reviewed for specific clinical manifestations and disease outcomes, including types of melioidosis (bacteremic, nonbacteremic, disseminated or localized), organs involved (lungs, liver, spleen, bone, soft tissues, brain and genitourinary) and death. All clinical definitions and classifications were categorized as mentioned by Zueter et al. [23]. Strain tropism and virulence were studied by displaying clinical outcomes throughout the phylogenetic tree topology prepared from the STs. On the other hand, all closely-related STs were gathered into groups and analyzed as independent variables (predictors) against clinical outcomes that were identified as dependent variables. Statistical analyses were performed to analyze each genotype cluster with every clinical outcome using Pearson’s chi-square or Fisher exact tests.

### Ethics statement

Ethical approval was obtained from the Universiti Sains Malaysia Research Ethics Committee (Human) (USM/JEPeM/15110495) and data were analyzed anonymously.

## Results

Of the 83 clinical *B. pseudomallei* isolates obtained in this study, 32 STs were identified. The frequencies of STs among the 83 isolates were 1–12 observations with a predominance of ST54 (*n* = 12), ST371 (*n* = 7) and ST289 (*n* = 7).

Among the obtained STs, the number of alleles per locus varied from 3 to 6. SNPs were observed at all seven loci, with the number of SNPs ranging from 2 to 21, while the number of polymorphic (variable) sites within the different alleles at the seven loci varied between 2 and 15. The levels of locus sequence diversity among all 32 STs were 2.5 to 5.3% (Table 1). All STs identified in this study were deposited in the MLST database with complete reference annotation (Table 2).

**Table 1:** Properties of the MLST loci in the clinical *B. pseudomallei* isolates from Peninsular Malaysia

Locus	No. of nucleotides analyzed	No. of alleles	No. of SNP	SNP Frequency^a^	No. of variable sites	Sequence diversity rate^b^
*Ace*	519	4	3	0.6%	3	4.1%
*gltB*	522	5	8	1.5%	3	3.1%
*gmhD*	468	5	12	2.5%	5	4.0%
*lepA*	486	6	21	4.3%	15	5.3%
*lipA*	402	5	7	1.7%	4	2.9%
*narK*	561	4	9	1.6%	5	3.3%
*Ndh*	443	3	2	0.5%	2	2.5%

^a^Rate of SNPs diversity in relation with locus length (no. of SNP/locus length)

^b^Rate of allele diversity in relation with the number of total referenced database alleles

**Table 2:** Properties of *B. pseudomallei* sequence types in this study

Isolate code	Origin (specimen)	Sequence type		
			Strain name	MLST database ID
2	Blood	54	USM2	3668
3	Blood	54	USM3	3669
7	Pus	54	USM7	3670
15	Body fluid	54	USM15	3671
69	Blood	54	USM69	4066
47	Pus	54	USM47	3672
48	Blood	54	USM48	3673
50	Blood	54	USM50	3674
43	Urine	54	USM43	3675
22	Body fluid	54	USM22	3676
27	Body fluid	54	USM27	3677
40	Blood	54	USM40	3678
8	Blood	371	USM8	3679
12	Blood	371	USM12	3718
14	Blood	371	USM14	3680
24	Blood	371	USM24	3681
33	Blood	371	USM33	3682
35	Blood	371	USM35	3683
71	Blood	371	USM71	4018
6	Pus	46	USM6	3684
45	Sputum	46	USM45	3685
20	Blood	46	USM20	3686
57	Blood	46	USM57	3687
32	Blood	46	USM32	3688
61	Pus	46	USM61	3689
39	Blood	84	USM39	3690
9	Pus	84	USM9	3691
28	Body fluid	84	USM28	3692
64	Blood	84	USM64	3693
42	Blood	289	USM42	3694
44	Blood	289	USM44	3695
49	Blood	289	USM49	3696
13	Blood	289	USM13	3697
5	Blood	289	USM5	3698
66	Pus	289	USM66	4016
63	Blood	289	USM63	3699
29	Blood	271	AMON29	3714
74	Blood	271	USM74	4025
78	Blood	271	USM78	4026
79	Blood	271	USM79	4027
36	Blood	306	USM36	3700
53	Pus	306	USM306	3701
58	Blood	306	USM58	3702
37	Pus	306	USM37	3703
10	Blood	55	USM10	3708
23	Blood	55	USM23	3709
18	Pus	50	USM18	3704
51	Sputum	50	USM51	3705
54	Blood	50	USM54	3706
41	Blood	50	USM41	3707
38	Blood	376	USM38	3710
17	Pus	376	USM17	3711
31	Pus	507	HANA31	3713
46	Blood	51	ZED46	3712
60	Body fluid	10	USM60	4015
67	Blood	164	USM67	4022
73	Blood	164	USM73	4023
80	Blood	164	USM80	4024
68	Blood	369	USM68	4017
72	Blood	402	USM72	4019
82	Blood	368	USM82	4021
75	Blood	47	USM75	4028
77	Blood	47	USM77	4029
81	Blood	47	USM81	4030
83	Pus	47	USM83	4031
76	Blood	168	USM76	4020
11	Blood	1319	11	3659
65	Blood	1319	USM65	4067
1	Blood	1317	1	3657
4	Blood	1318	4	3658
19	Blood	1320	19	3660
21	Blood	1321	21	3661
25	Body fluid	1322	25	3662
26	Body fluid	1322	AMAR26	3715
30	Pus	1323	30	3663
16	Blood	1323	USM16	4014
34	Blood	1324	34	3664
52	Body fluid	1325	52	3665
55	Blood	1326	55	3666
56	Blood	1326	HAMZ56	3716
59	Blood	1327	59	3667
62	Pus	1358	ABD12	4032
70	Body fluid	1359	NOR13	4033

### Genetic relatedness among studied B. Pseudomallei sequence types

**Fig. 1:**
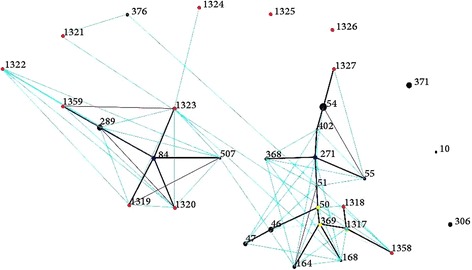
eBURST diagram representing the relatedness between 32 STs identified in 83 isolates. Black dot: existing ST. Red dot: novel ST. Blue dot: predicted group ancestor. Yellow dot: predicted subgroup ancestor. Green dot: novel and subgroup ancestor ST. Black and purple lines: single locus variants (SLVs). Blue line: double locus variant (DLV). Re-samplings for bootstrapping = 10,000; minimum number of identical loci for group definition =6; minimum number of SLV for subgroup definition =3. The size of the dot reflects the individual ST frequency among the 83 strains

Half of the STs were clustered into a single group of 16 STs, of which four were novel (Fig. 1). The STs were presented in 44 isolates clustered into a major group and emerged from ST271 representing the predicted founder. An additional three subgroup founders branched from ST271 were also identified including ST50, ST369 and ST1317. ST84 was the predicted as ancestor to another smaller population group consisting of six STs, and most were novel. The remaining STs were singletons.

### Genetic relatedness among B. Pseudomallei sequence types in Malaysia

Thirteen STs identified in this study were novel, including ST1317, ST1318, ST1319, ST1320, ST1321, ST1322, ST1323, ST1324, ST1325, ST1326, ST1327, ST1358 and ST1359. On the other hand, the other STs (*n* = 19) reported in this study were also characterized elsewhere in the Indian subcontinent, China and Southeast Asia.

**Fig. 2:**
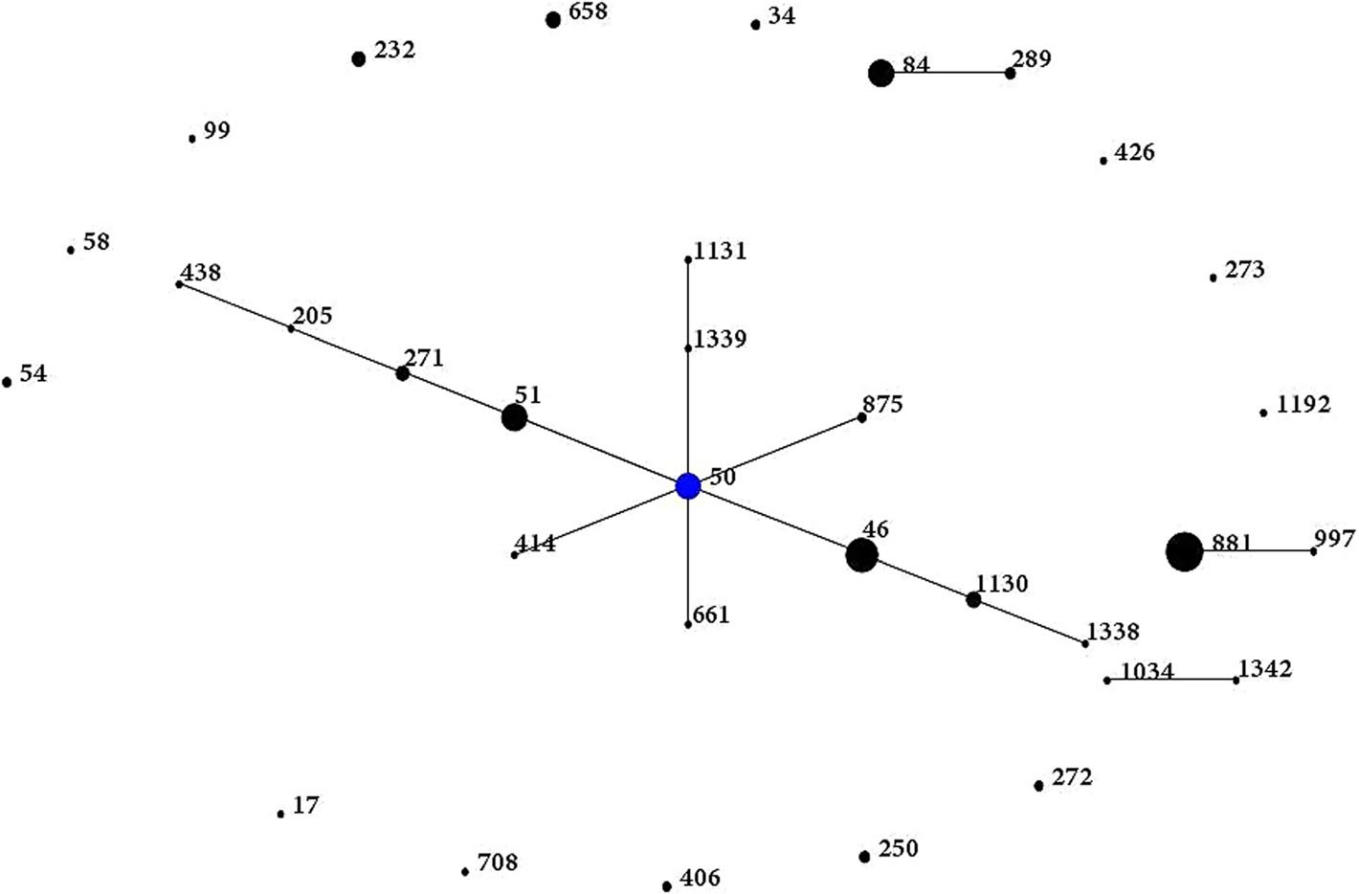
eBURST population snapshot for *B. pseudomallei* STs in Malaysia before conducting the present study. Blue dot refers to group founder. Each black dot represents single genotype. The size of the dot represents the ST frequency

**Fig. 3:**
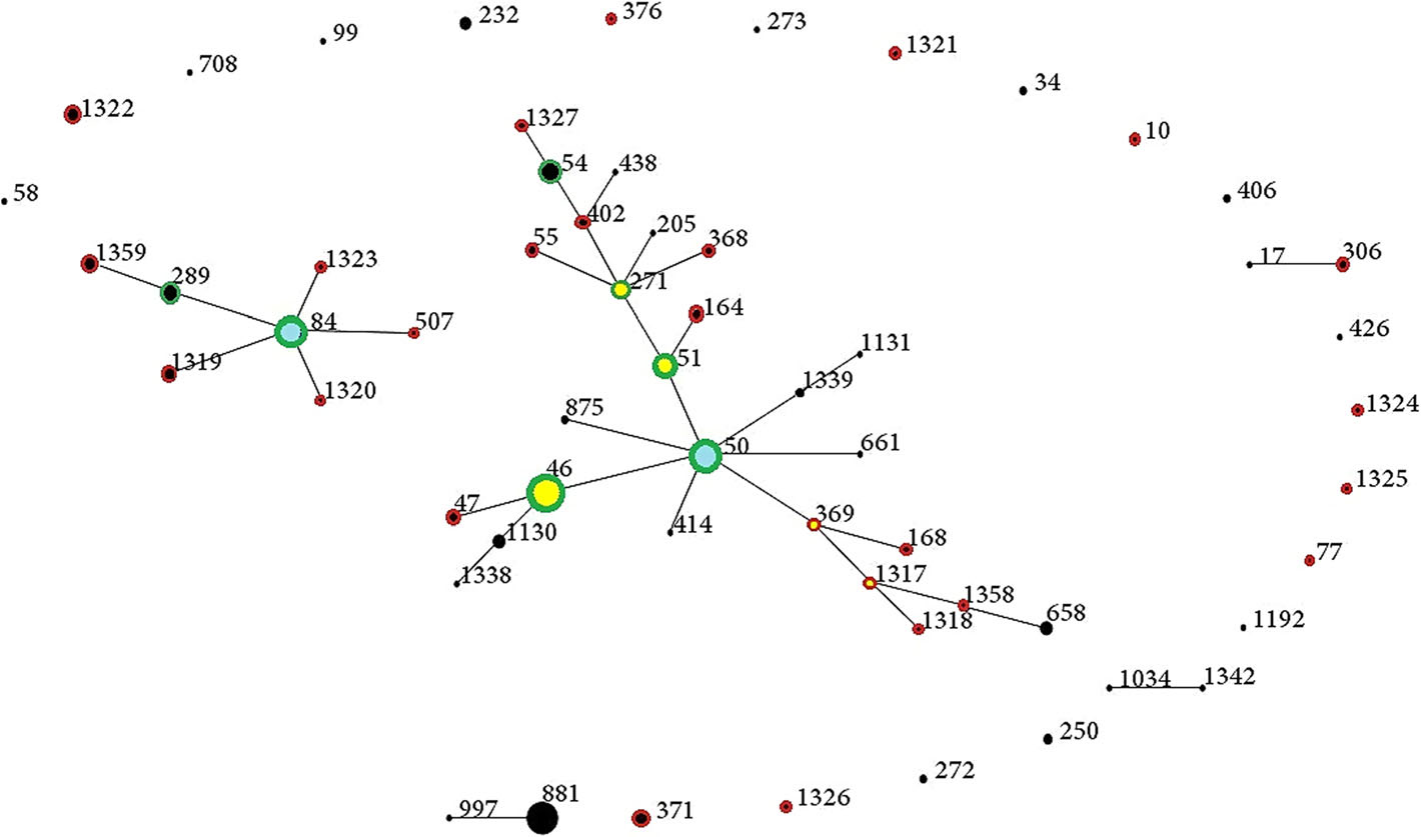
Overall *B. pseudomallei* STs in Malaysia showing STs added by this study. Black dot: ST only in Malaysian database query. Red hollow: ST only in this study. Green hollow: ST in both Malaysian query and present study. Yellow dot: subgroup founder. Blue dot: Group founder. Re-samplings for bootstrapping = 10,000; minimum number of identical loci for group definition =6; minimum number of SLV for subgroup definition =3. The size of the dot reflects the individual ST frequency among the 83 strains

Total of 264 *B. pseudomallei* isolates and 59 STs were already registered in the database (MLST.net) until April 2015, all of which were from Malaysia. The present study uploaded additional 83 *B. pseudomallei* isolates and 32 STs from the same country. Before the present study, almost half of Malaysian STs were clustered into a single group with ST50 as the predicted founder. The remaining STs were singletons. No sub-groups were reported (Fig. 2). The present study has expanded the former Malaysian clonal cluster by adding more branching STs. In addition, new clonal expansion has emerged from ST84 to create another group in the Malaysian database (Fig. 3). This expansion was characterized by conversion of ST84 from an existing ST into a new ancestral group founder from which other single and double locus variant STs have emerged. In addition, another sub-clonal expansion was created from ST51, ST271, ST46, ST369 and ST1317.

### Phylogenetic relationship among regional B. Pseudomallei sequence types

**Fig. 4:**
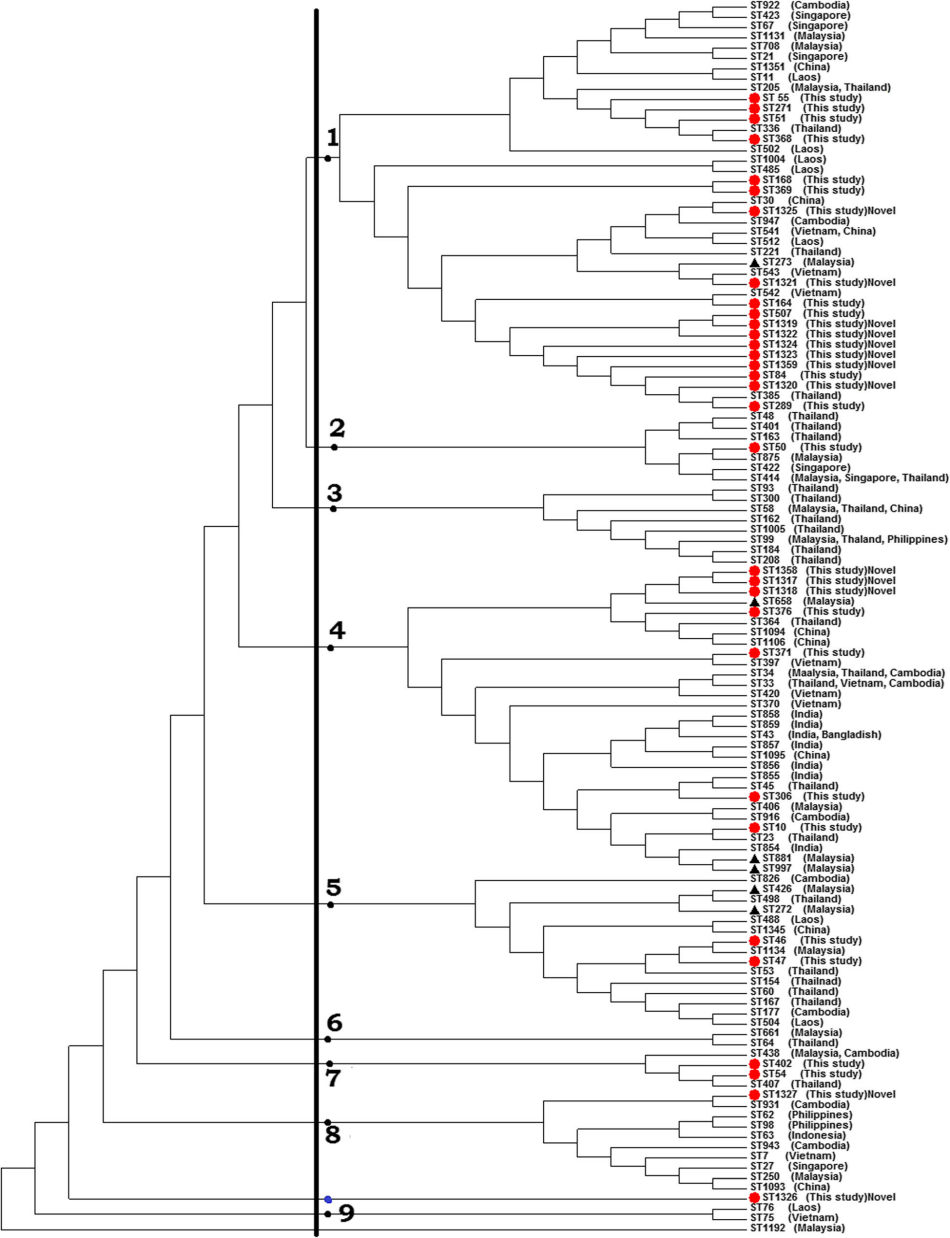
The evolutionary history inferred using the UPGMA method to analyze the studied 32 STs along with 88 historical STs represented India, China and Southeast Asian countries.▲: Sarawak ST

The majority of the STs formed unique sequences that differed by at least a single nucleotide and almost all were seen in all groups in the phylogenetic tree (Fig. 4). More than half of the group 1 STs were clustered with each other, as well as with STs from Malaysia, Thailand, Singapore, Cambodia, Vietnam, Laos and China. On the other hand, ST50 and the novel ST1327 were not grouped with any of our STs but were clustered with local STs and with narrower regional STs located in groups 2 and 8, respectively. The remaining STs were distributed among other groups with little distance between them. The STs in the lower sub-cluster of group 4 and in group 5 were clustered with STs that have been reported from Sarawak in West Malaysia. The majority of the novel STs were clustered with each other in any given group. Of the 13 novel STs, eight were located in group one. The only unique ST in this study was ST1326, which was novel and a singleton.

### *B. pseudomallei* genotype - disease associations

**Fig. 5:**
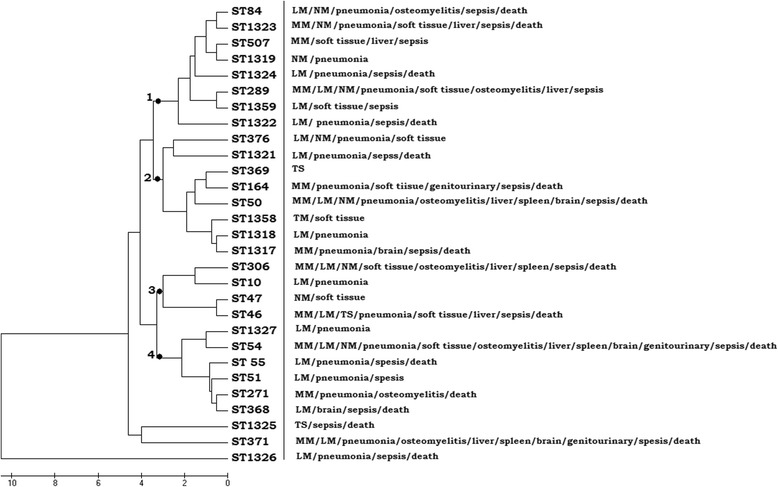
Topology of clinical outcomes on phylogenetic tree. MM: multifocal melioidosis, LM: localized melioidosis, NM: nonbacteremic melioidosis, TS: transient septicemia. 1: first genotype cluster; 2: second genotype cluster; 3: third genotype cluster; 4: fourth genotype cluster

The clinical histories of 70 subjects in whom bacterial genotypes were identified and archived were reviewed from 2007 to 2014. No evidence supporting an association between *B. pseudomallei* STs and any clinical presentation of melioidosis was observed on the phylogenetic tree; no clustering was noted for a given clinical outcome with a particular genotype (Fig. 5).

In addition, no evidence of differential virulence or strain tropism was detected. For example, severe sepsis (*n* = 11) was caused by strains of seven different STs, whereas septic shock (*n* = 29) and abscess (*n* = 30) were caused by strains of 17 and 18 different STs, respectively.

The two-way tables for all bacterial genotype clusters in relation to clinical outcome variables were statistically non-significant (*p* > 0.05), with no reported risk estimate for any genotype cluster developing any of the clinical outcome (data not shown).

## Discussion

*Burkholderia pseudomallei* is Gram negative saprophytic bacterium classified as Tier 1 Biological Select Agent [24]. Due to frequent recombination, the *B. pseudomallei* genome showed high plasticity that increases genetic divergence, and therefore strain-to-strain variation [25]. The spectrum of *B. pseudomallei* genetic diversity in Peninsular Malaysia and its association with clinical outcomes is not yet known. It is therefore important to determine ST genotypes to compare the molecular epidemiology of *B. pseudomallei* in Peninsular Malaysia with strains obtained from other regions, especially other countries within Asia, and to investigate genotype diversity as a possible explanation for differences in disease presentation, treatment response, prognosis and mortality [26].

In the present study, STs were identified with different frequencies, predominance, novelty, and allelic heterogeneity. The overall diversity of isolates found in the clinical specimens was 0.38 STs/isolate, compared with a diversity ratio of 0.65 STs/isolate reported in Australia.^26^ Several molecular studies that applied various genotyping methods to clinical *B. pseudomallei* isolates reported genotypic novelty and diversity with or without predominance of particular genotypes among single population communities of temperate endemic areas of Malaysia [10, 15, 27–29], Thailand [9, 30], India [31], and Australia [2, 32].

The presence of different genotypes with various frequencies reflects the historical introduction and dissemination of different *B. pseudomallei* genotypes into the study area or due to expansion of local STs that yielded new strains with novel STs [33]. Genotypic predominance might be attributed to localization of a particular genotype in the study area in which the contaminated environment became a rich source for infection by that genotype [34]. For example, the predominant STs found in this study were ST54, ST371, ST46 and ST84, which have been found in Malaysia and neighbouring countries. Moreover, some genotypes identified in this study such as ST402, ST55, ST271, ST376, ST47, and ST376, have been identified in soil and water sources in Malaysia and other neighboring countries [19, 33].

This genotypic picture for our clinical isolates might be linked to the endemic geographical distribution of *B. pseudomallei* in the environments our patients resided. This suggestion is supported by reports of melioidosis outbreaks caused by *B. pseudomallei* of the same genotypes as those of the suspected environmental sources [16, 35–38].

The presence of novel genotypes indicates local persistence of *B. pseudomallei* in the same geographical area and their ability to establish a new clone series producing novel offspring’s that carry new genotypes [39]. Several reports have documented the emergence of novel *B. pseudomallei* genotypes regardless of the number of the genotyped isolates [15, 31]. In this study, two of 10 strains isolated from patients residing in Bachok were novel genotypes, whereas 3/15 (20%), 1/10 (10%), 2/8 (25%) and 2/4 (50%) strains carried novel genotypes in Terengganu, Selangor, Pasir Puteh and Machang, respectively.

The characteristics of the alleles and loci were considerably diverse among the 32 STs. However, no new alleles have been reported. Previous studies suggested a high rate of recombination replacement relative to substitution mutations in *B. pseudomallei* that caused re-assortment of existing alleles, rather than emergence of new alleles, leading to a new generation of STs [32, 39].

The changes occurring in ST84 (as seen in the eBURST snapshots) before and after this study suggest the occurrence of clonal expansion of ST84. This conclusion was reached based on the presence of seven novel STs arising from ST84 and would be supported by confirming the evolutionary convergence of ST84 from a singleton ST to the group founder ST. In the same way, other sub-clonal expansions were created from ST51, ST271, ST46, ST369, and ST1317. Thus, the present study has markedly expanded the former Malaysian clonal cluster by adding more branching STs.

McCombie et al. [33] had studied the molecular epidemiology of *B. pseudomallei* using MLST of 207 historical isolates collected in Malaysia, Thailand and Vietnam. MLST revealed 80 STs and 56 were novel. When those STs were added to the *B. pseudomallei* MLST database and analyzed together, the historical-collection STs clustered significantly within the complex of the eBURST diagram in an ancestral pattern and expanded the *B. pseudomallei* population snapshot. In the same study, ST84 was likely a *B. pseudomallei* isolate characteristic of Southeast Asia rather than Australia based on abundance in several environmental isolates from Thailand and Malaysia.

Clustering of our STs in the phylogenetic tree with STs from Sarawak, Thailand, Singapore, Cambodia, Vietnam, Laos and China suggests their genetic relatedness with ST ancestors of these regions. In addition, all non-novel STs identified in this study were also identified in these countries at different frequencies, which suggesting that the Malaysian isolates may not be distinct from those of Southeast Asia. ST371, ST164, ST47, ST306, ST55, ST376, ST402, ST507, ST368, ST369, ST10 and ST168 were first identified in Malaysia. Nevertheless, these STs are not found exclusively in Malaysia only but also in other Southeast Asian countries. This topology explores the geographical expansion and spread of those STs among regional countries through environmental and human routes [32]. Such expansion was restricted to countries bordering with Malaysia but not other regions, such as Australia, Africa, or Latin America, due to the absence of shared STs with those regions, which concurs with previous findings of no shared STs among different continents. However, a few exceptions have been more recently reported; in one study, ST105 and ST849 were shared STs between Australia and Cambodia and both STs were isolated from patients from both countries [40]. Another study reported the isolation of ST562 from Australia and China [41].

Clinical outcome-genotyping association in human cases has not been clearly described in Malaysia and interpretative studies on the significance of genotyping results remain limited. In this study, tests to cluster clinical presentation on the phylogenetic tree, differential virulence tropism for an individual ST, and statistical associations between genotype clusters with clinical presentations did not detect any relationship between genotype and disease. Two Australian studies genotyped clinical isolates of *B. pseudomallei* using PFGE and MLST. The clinical history of each patient was reviewed and analyzed statistically in combination with the resulting genotypes. However, neither study found an association due to the high diversities of the genotypes and clinical presentations and low relative frequencies of each of them. In addition, no association was reported between a given genotype and a particular clinical presentation or site of infection [2, 26]. On the other hand, a study from Thailand reported partial and possible associations between *B. pseudomallei* ribotypes and clinical outcomes of melioidosis. However, that study was not conclusive due to low number of tested cases [11]. Our study concurs with the previous studies demonstrating a lack of an association between any ST and disease, but considers that host and environmental factors are reasons for the heterogenous nature of the clinical presentation of the disease.

## Conclusion

The present study revealed the high diversity of *B. pseudomallei* in Malaysia, and several STs were discovered. Many of the non-novel STs found in this study were also reported from neighboring Asian countries. None of the STs were associated a specific disease presentation. Therefore, host and environmental factors play crucial roles in the diversity of clinical presentation and outcomes of the disease. Further studies on environmental samples (and a comparison with clinical isolates) may provide more extensive, representative data to elucidate the course and evolution of the *B. pseudomallei* population in this region. Expanding the clinical case review would provide more data for further understanding of specific genotype-disease association in melioidosis.

## Abbreviations

MEGA: Molecular evolutionary genetics analysis
MLST: Multi-locus sequence typing
PCR: Polymerase chain reaction
PFGE: Pulsed-field gel electrophoresis
RAPD: Random amplification of polymorphic DNA
SNP: Single nucleotides polymorphism
ST: Sequence type
UPGMA: Unweighted Pair Group Method with Arithmetic average
